# Posterior division of ipsilateral C7 transfer to C5 for shoulder abduction limitation

**DOI:** 10.3389/fneur.2023.1012977

**Published:** 2023-02-02

**Authors:** Xinying Huang, Zongqi You, Yaoxian Xiang, Junxi Dai, Junjian Jiang

**Affiliations:** ^1^Department of Hand Surgery, Huashan Hospital, Fudan University, Shanghai, China; ^2^Key Laboratory of Hand Reconstruction, Ministry of Health, Shanghai, China; ^3^Shanghai Key Laboratory of Peripheral Nerve and Microsurgery, Shanghai, China; ^4^Shanghai Medical College, Fudan University, Shanghai, China

**Keywords:** nerve transfer, ipsilateral C7 transfer, brachial plexus injuries, neurogenic shoulder abduction limitation, C5 injury

## Abstract

**Background:**

Reparation of C5 by proximal selective ipsilateral C7 transfer has been reported for the treatment of neurogenic shoulder abduction limitation as an alternative to the reparation of the suprascapular nerve (SSN) and the axillary nerve (AXN) by distal nerve transfers. However, there is a lack of evidence to support either strategy leading to better outcomes based on long-term follow-up.

**Objective:**

The purpose of the study was to investigate the safety and long-term outcomes of the posterior division of ipsilateral C7 (PDIC7) transfer to C5 in treating neurogenic shoulder abduction limitation.

**Methods:**

A total of 27 cases with limited shoulder abduction caused by C5 injury (24 cases of trauma, 2 cases of neuritis, and 1 case of iatrogenic injury) underwent PDIC7 transfer to the C5 root. A total of 12 cases (11 cases of trauma and 1 case of neuritis) of C5 injury underwent spinal accessory nerve (SAN) transfer to SSN plus the triceps muscular branch of the radial nerve (TMBRN) transfer to AXN. The patients were followed up for at least 12 months for muscle strength and shoulder abduction range of motion (ROM).

**Results:**

In cases that underwent PDIC7 transfer, the average shoulder abduction was 105.9° at the 12-month follow-up. In total, 26 of 27 patients recovered at least M3 (13 reached M4) (Medical Research Council Grading) of the deltoid. In cases that underwent SAN transfer to SSN plus TMBRN to AXN, the average shoulder abduction was 84.6° at the 12-month follow-up. In total, 11 of 12 patients recovered at least M3 (4 reached M4) of the deltoid.

**Conclusion:**

Posterior division of ipsilateral C7 transfer is a one-stage, safe, and effective surgical procedure for patients with neurogenic shoulder abduction limitation.

## Introduction

Neurogenic shoulder abduction limitation, a pathological change of the nerves innervating shoulder abductors, can be caused by C5 disorder. C5 contributes to the axillary nerve (AXN), which dominates the deltoid, and the suprascapular nerve (SSN), which dominates the musculus supraspinatus and the musculus infraspinatus (1). Based on our clinical experiences and previous studies, trauma, especially traffic accidents, is the most common cause leading to the direct injury of C5. However, the direct injury of C5 can also be caused by other neurological reasons, such as neuritis, peripheral neuropathy, and iatrogenic injury, in some patients. Those patients were treated with conservative therapies such as drugs or physical therapies, but they received poor results. In a retrospective study reported by Thompson et al. (2), among 59 patients with postoperative C5 disorder who received conservative therapies, 15 patients had symptoms resolved with residual effects (25.4%) and 10 patients did not recover (17.0%).

Nerve transfer strategies have been reported to mainly focus on the distal nerve transfer as repairing SSN and AXN separately since they are the main branches of the C5 nerve root and their targeting muscles are involved in shoulder movement. Surgeons are used to choosing the donor nerves outside the brachial plexus such as accessory nerve (3, 4) and phrenic nerve (5). However, several issues with previous strategies need to be addressed. First, muscle function recovery requires adequate motive power from the donor nerve, matching donor nerve, and the recipient's nerve. It is a dilemma that choosing a donor nerve with sufficient motive power may lead to a severe loss of their original target muscle function such as diaphragm disorder caused by phrenic nerve transfer. Another drawback of previous strategies focused on distal nerve transfer is that C5 injury cannot be directly diagnosed without an additional incision to explore the brachial plexus. For intraoperative diagnosis, a specific incision for brachial exploration is required, resulting in more surgical injury and even a two-stage surgical strategy. Furthermore, another problem is that if the cerebral cortex regions of the donor's nerve and recipient's nerve are distant, it could be more difficult for patients to voluntarily move after surgery and the recovery process places high demands on brain plasticity (6).

Since distal nerve transfer may lead to the problems mentioned earlier, Gu et al. (7) developed ipsilateral C7 nerve root transfer to treat C5 rupture and Xu et al. (8, 9) further developed selective ipsilateral C7 nerve root transfer with less donor nerve function loss. To investigate further, here, we studied the clinical outcomes of 27 patients with the posterior division of ipsilateral C7 (PDIC7) transfer, a more selective proximal C5 repair strategy compared to distal SSN and AXN repair strategies for the treatment of neurogenic shoulder abduction limitation.

## Methods

### Patients

This is a retrospective study carried out in accordance with the Declaration of Helsinki. Each participant provided informed written consent. IRB approval was granted by our institution. Based on the following criteria, we compared 27 patients who underwent PDIC7 transfer to C5 with 12 patients who underwent SAN transfer to SSN plus TMBRN transfer to AXN.

The inclusion criteria are as follows:

1. Patients with shoulder abduction limitation whose muscle strength of the musculus supraspinatus and deltoid was M0.
2. C5 injury which was diagnosed by neurophysiological investigations and ultrasound examination and then confirmed by subsequent intraoperative exploration and neurophysiological investigation.
3. Reserved C7 function was diagnosed by the neurophysiological investigations and confirmed by subsequent intraoperative exploration and neurophysiological investigation.

The exclusion criteria are as follows:

1. History of diabetic mellitus and smoking history.
2. Fractures of the affected limb, neuromuscular disorders, and musculoskeletal disorders such as tendon or ligament injury were excluded.
3. Cases with an interval time between injury and surgery of less than 1 month or more than 12 months.

### Surgical procedures

#### PDIC7 transfer to C5

The operation was carried out under general anesthesia without using muscle relaxants. The arm, shoulder, neck, and chest were prepared with the patient supine.

**Figure 1:**
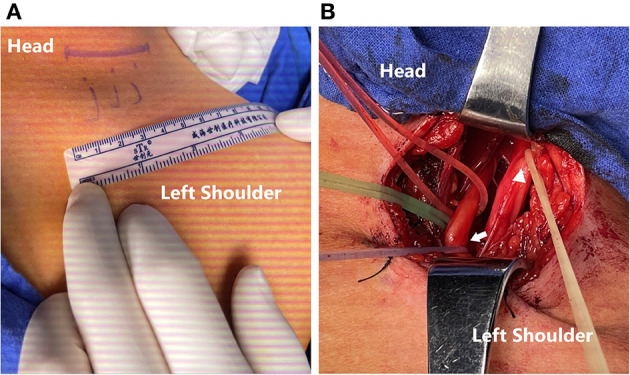
**(A)** An incision for around 4 cm-length centered over the clavicle. **(B)** Exploration of the brachial plexus and an intraoperative EMG test to reconfirm that the C5 root (yellow line; triangle) had been avulsed and the C7 root (red line), especially PDIC7 (purple line; arrow) had normal electrophysiological function.

**Figure 2:**
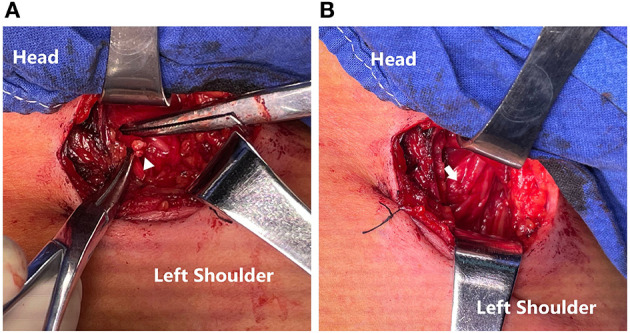
**(A)** Ends of PDIC7 and C5 root (triangle). **(B)** Transfer of the PDIC7 to the C5 root (arrow).

The brachial plexus was explored through an incision of around 4 cm in length centered over the clavicle (Figure 1A), followed by an exploration of the anatomical structure of the 5 roots (Figure 1B). The continuity of the upper, middle, and lower trunks of the brachial plexus was then confirmed. C5 injury was identified by the absence of sensory-evoked potential (SEP) and compound muscle action potential (CMAP) of the deltoid while C6-T1 roots were unaffected. CMAP of the triceps and latissimus dorsi identified PDIC7. Using 8–0 sutures under a magnification of 2.5x, the PDIC7 was transferred to the C5 root (Figure 2).

#### SAN transfer to SSN plus TMBRN transfer to AXN

The operation was carried out as a two-stage procedure performed under general anesthesia without using muscle relaxants.

In the first stage, the brachial plexus was explored through an incision centered over the clavicle. The continuity of the upper, middle, and lower trunks of the brachial plexus was then confirmed. C5 injury was identified by the absence of sensory-evoked potential (SEP) and compound muscle action potential (CMAP) of targeted muscles. The SSN was identified from the injured C5 root and the SAN was also separated in the same incision. The SAN was transferred to SSN. Additionally, in the second stage, the TMBRN was separated and transferred to AXN through a longitudinal incision on the posterior aspect of the arm. Details of the surgical procedures have been described before (10).

### Postoperative management

Patients were asked to use a custom-made neck splint postoperatively for 3 weeks. Physiotherapy began 4 weeks after surgery to maintain the ROM in all joints, such as shoulder abduction, external rotation, and elbow flexion. However, passive shoulder abduction beyond 90° was avoided within 4 weeks after nerve repair.

### Postoperative evaluation

Postoperative evaluations were performed at 14 days and then at approximate intervals of 3 months, with at least 12 months until no improvement can be observed.

A physical assessment and an electromyographic test were conducted in the clinic. The motor function was classified according to the Medical Research Council grading. The active and passive ranges of external rotation were evaluated beginning with the arm in the neutral position. The sagittal plane was defined as 0°. The range of movement was defined as the angle between the 0 and 180° position and the forearm position upon an external abduction (9).

Electromyography was conducted 3 months after surgery. CMAP of the deltoid was recorded as the sign of successful C5 regeneration and CMAP of the latissimus dorsi was recorded for evaluating the function loss of PDIC7.

### Statistical analysis

Outcomes comprising continuous variables such as ROM were presented as mean ± std and analyzed using the *t*-test. The *p*-values were two-tailed and *p-*values of <0.05 were considered significant. All statistical analyses were conducted using SPSS for Windows.

## Results

### The characteristics of patients

From 2018 to 2020, 27 patients, 23 men, and 4 women, aged 16 to 63 years (average, 38.7 years), had brachial plexus C5 injury, and the average interval between injury and surgery was 3.9 months. A total of 12 patients were investigated in the group of PDIC7 transfer to C5. A total of 11 men and 1 woman, aged 36–67 years (average, 45.6 years), were investigated in the group of SAN transfer to SSN plus TMBRN transfer to AXN, and the average interval between injury and surgery was 4.1 months.

### Motor and sensory function of the donor site

During the follow-up period, no patient in PDIC7 to C5 was observed with obvious elbow extension disorder or upper extreme adduction and pronation disorder. For PDIC7, we focused on the function of the triceps and latissimus dorsi with manual muscle testing, and reversible muscle strength decrease within 1 level (more than M3 postoperation) was thought to be acceptable. The follow-up EMG did not show the severe loss of function of the triceps and the latissimus dorsi, which showed that the motor function of the PDIC7 can be compensated. In terms of sensory function, 2 patients complained of transient middle finger numbness, 1 patient disappeared spontaneously during the follow-up period, and 1 patient with persistent numbness could adjust the discomfort by himself, and it was considered that it did not affect the quality of life.

For patients who underwent SAN transfer to SSN plus TMBRN transfer to AXN, we also tested the trapezius muscle and triceps in SAN transfer to SSN plus TMBRN transfer to AXN with the same standard as PDIC7. There were no evident motor or sensory function disorders in the dominated area of the donor's nerve, and the EMG test showed acceptable nerve and muscle function of the radial nerve and triceps.

### Recovery of shoulder function

**Figure 3:**
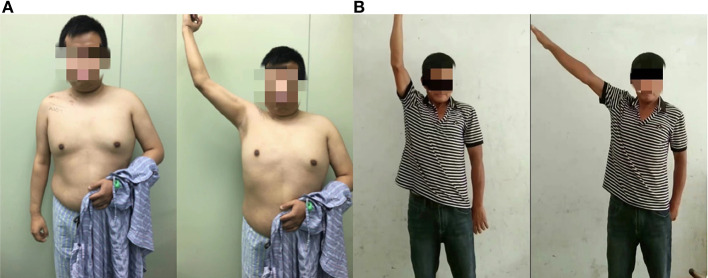
**(A)** Patient's functional recovery at month. **(B)** Patient's functional recovery at 2 years.

In all 27 patients who underwent PDIC7 to C5, the average maximal range of shoulder abduction was 105.9° from the worst case 65° to the best case 170° (Figure 3). A total of 26 of 27 patients achieved at least M3 muscle strength within 12 months and 50% of cases achieved M4 level. Among those cases of M3, 7 of 13 patients' injured side was not the dominant side. In contrast, in 12 patients who underwent SAN transfer to SSN plus TMBRN transfer to AXN, the average maximal range of shoulder abduction was 84.6° from the worst case 50° to the best case 100° and 11 of 12 patients achieved at least M3 muscle strength within 12 months and 36.4% cases achieved M4 level. The maximal range of shoulder abduction in patients who underwent PDIC7 to C5 was significantly greater than those who underwent SAN transfer to SSN plus TMBRN transfer to AXN (*p* < 0.05, Table 1).

**Table 1:** *t*-test result of the maximal range of shoulder abduction in two groups.

**Method (Mean ±*s*)**	**ROM**
PDIC7 (*n* = 27)	105.93 ± 27.18
SAN-SSN + TMBRN-AXN (*n* = 12)	84.58 ± 14.05
*t*	3.224
*p*	0.003^**^

ROM, Range of motion. ^**^p < 0.01.

All the detailed information of patients in our study is listed in Table 2.

**Table 2:** Detailed information on patients who underwent one of the two types of surgeries.

**Sex**	**Age**	**Dominant side**	**Injured side**	**Cause**	**Surgery**	**Interval between injury and surgery**	**Duration of follow-up /months**	**Active ROM of shoulder abduction improvement**	**Shoulder abduction strength improvement**	**Preoperative EMG (deltoid)**
Male	27	Right	Left	surgical injury	PDIC7—C5	2	12	0–90	M1-M3	MUP(–), CMAP(–)
Male	35	Left	Left	traffic accident	PDIC7—C5	2	12	0–100	M1-M4	MUP(–), CMAP(–)
Male	51	Right	Right	traffic accident	PDIC7—C5	3	24	0–100	M1-M4	MUP(–), CMAP(–)
Male	31	Right	Right	traffic accident	PDIC7—C5	3	12	0–90	M1-M4	MUP(–), CMAP(–)
Male	23	Right	Right	traffic accident	PDIC7—C5	2	12	0–170	M1-M4	MUP(–), CMAP(–)
Female	46	Right	Left	crush injury	PDIC7—C5	4	12	0–80	M1-M3	MUP(–), CMAP(–)
Male	37	Right	Right	neuritis	PDIC7—C5	6	12	0–95	M1-M3	MUP(–), CMAP(–)
Male	63	Right	Right	traffic accident	PDIC7—C5	3	12	0–80	M1-M3	MUP(–), CMAP(–)
Male	52	Right	Left	traffic accident	PDIC7—C5	3	30	0–80	M1-M3	MUP(–), CMAP(–)
Male	47	Right	Right	traffic accident	PDIC7—C5	4	36	0–120	M1-M4	MUP(–), CMAP(–)
Female	42	Right	Right	traffic accident	PDIC7—C5	4	12	0–130	M1-M4	MUP(–), CMAP(–)
Male	19	Right	Left	traffic accident	PDIC7—C5	3	12	0–120	M1-M4	MUP(–), CMAP(–)
Male	20	Right	Left	traffic accident	PDIC7—C5	5	12	0–90	M1-M3	MUP(–), CMAP(–)
Male	55	Right	Right	traffic accident	PDIC7—C5	8	30	0–90	M1-M3	MUP(–), CMAP(–)
Female	34	Right	Left	traffic accident	PDIC7—C5	2	12	0–90	M1-M3	MUP(–), CMAP(–)
Male	54	Right	Right	falling injury	PDIC7—C5	2	12	0–100	M1-M4	MUP(–), CMAP(–)
Male	31	Right	Right	traffic accident	PDIC7—C5	6	12	0–90	M1-M3	MUP(–), CMAP(–)
Male	47	Left	Right	traffic accident	PDIC7—C5	9	12	0–65	M1-M2	MUP(–), CMAP(–)
Male	52	Right	Left	traffic accident	PDIC7—C5	5	12	0–100	M1-M3	MUP(–), CMAP(–)
Male	16	Right	Left	neuritis	PDIC7—C5	7	12	0–95	M1-M3	MUP(–), CMAP(–)
Male	29	Right	Right	falling injury	PDIC7—C5	3	12	0–155	M1-M4	MUP(–), CMAP(–)
Male	45	Right	Right	strangulation	PDIC7—C5	6	21	0–100	M1-M4	MUP(–), CMAP(–)
Male	54	Right	Right	crush injury	PDIC7—C5	1	27	0–165	M1-M4	MUP(–), CMAP(–)
Male	51	Right	Right	falling injury	PDIC7—C5	1	12	0–100	M1-M3	MUP(–), CMAP(–)
Female	26	Right	Left	incised injury	PDIC7—C5	2	12	0–125	M1-M4	MUP(–), CMAP(–)
Male	63	Right	Right	crush injury	PDIC7—C5	4	24	0–90	M1-M3	MUP(–), CMAP(–)
Male	16	Right	Left	surgical injury	PDIC7—C5	3	12	0–150	M1-M4	MUP(–), CMAP(–)
Male	51	Right	Left	crush injury	SAN-SSN TMBRN-AXN	7	12	0–50	M1-M2	MUP(–), CMAP(–)
Male	36	Right	Right	traffic accident	SAN-SSN TMBRN-AXN	1	12	0–80	M1-M3	MUP(–), CMAP(–)
Male	36	Right	Left	traffic accident	SAN-SSN TMBRN-AXN	8	12	0–95	M1-M3	MUP(–), CMAP(–)
Male	45	Right	Left	neuritis	SAN-SSN TMBRN-AXN	7	12	0–90	M1-M3	MUP(–), CMAP(–)
Male	39	Right	Right	crush injury	SAN-SSN TMBRN-AXN	2	12	0–90	M1-M4	MUP(–), CMAP(–)
Male	32	Right	Right	crush injury	SAN-SSN TMBRN-AXN	5	12	0–80	M1-M3	MUP(–), CMAP(–)
Male	42	Right	Left	crush injury	SAN-SSN TMBRN-AXN	2	12	0–90	M1-M3	MUP(–), CMAP(–)
Male	53	Right	Left	crush injury	SAN-SSN TMBRN-AXN	2	12	0–100	M1-M4	MUP(–), CMAP(–)
Male	56	Right	Right	falling injury	SAN-SSN TMBRN-AXN	3	12	0–70	M1-M3	MUP(–), CMAP(–)
Male	33	Right	Right	traffic accident	SAN-SSN TMBRN-AXN	2	12	0–100	M1-M4	MUP(–), CMAP(–)
Male	57	Right	Right	traffic accident	SAN-SSN TMBRN-AXN	5	12	0–90	M1-M4	MUP(–), CMAP(–)
Female	67	Right	Right	traffic accident	SAN-SSN TMBRN-AXN	5	12	0–80	M1-M3	MUP(–), CMAP(–)

## Discussion

In our study, we demonstrate a novel surgical strategy, posterior division of ipsilateral C7 (PDIC7) transfer, for the treatment of neurogenic shoulder abduction limitation and compared its effect on improving shoulder function with a widely-accepted surgical strategy, the spinal accessory nerve (SAN) transfer to the suprascapular nerve (SSN) plus TMBRN transfer to AXN.

Lu et al. (11) have explored the motor fiber counts of the human C7 roots and their branches. They showed that the posterior division of C7 owned 66.2% of all the motor fibers from the C7 root and these fibers finally joined to the axillary nerve (27.94%), radial nerve (49.75%), and thoracodorsal nerve (52.7%). Lu et al. (12) also observed in a rat model that the functions of C7 dominated muscles, including the triceps brachii and latissimus dorsi, can be compensated in 2 months after ipsilateral C7 transfer procedures. In conclusion, previous studies supported at the anatomic level that PDIC7 transfer could provide sufficient motive power for functional improvement without apparent donor deficits. In this study, we revealed that PDIC7 transfer may be an effective surgical strategy to improve shoulder function in cases of C5 injury. Also, all cases receiving PDIC7 transfer showed acceptable triceps brachii and latissimus dorsi function in EMG during follow-up.

Previous studies reported cross-sectional area (CSA) of the human brachial plexus in ultrasonography. Niu et al. (13) reported that the CSA of the C5 root was 5.3 ± 1.3 mm^2^ and of the C7 root was 8.5 ± 1.3 mm^2^. Similarly, Bedewi et al. (14) reported that the CSA of the C5 root at the interscalene groove was 5.1 ± 1.8 mm^2^ and of the C7 root was 6.3 ± 3.4 mm^2^. Won et al. (15) reported that the CSA of the C5 root was 5.66 ± 1.02 mm^2^ and of the C7 root was 10.43 ± 1.86 mm^2^. In our study, we observed that the CSA of PDIC7 and C5 roots matched well during operation. Also, as the donor nerve and acceptor nerve are close enough, the tension at the suture site can be small, which is beneficial for nerve regeneration.

Compared with SAN transfer to SSN plus TMBRN transfer to AXN, a widely-accepted surgical strategy, PDIC7 transfer to C5, has several advantages. First, surgeons could use a single supraclavicular incision for both brachial plexus exploration and repair. It is necessary to clarify the diagnosis of C5 injury during operation because C5 neurolysis alone may be sufficient to restore shoulder abduction if the continuity of C5 is reserved, whereas distal nerve transfer may lead to iatrogenic injury of SAN and TMBRN. Supportive evidence from Chuang et al. (16) showed that in their series, 9.5% of cases were identified intraoperatively as ruptured spinal nerves despite the preoperative imaging indicating complete root avulsion. Moreover, SAN transfer to SSN plus TMBRN transfer to AXN can only repair two nerves while PDIC7 transfer is to repair at the root level, which targets a group of muscles and can provide more motive power. Patients in the SAN-to-SSN plus TMBRN-to-AXN group did not experience sensory deficit, which is indeed a benefit of peripheral nerve transfer due to the presence of somatosensory fibers in either SAN or TMBRN. Although the patients in the PDIC7 group experienced sensory deficits, the sensory deficit was transient and not severe, which did not obscure the better motor recovery ability of PDIC7 transfer.

Another vital issue that should be considered for nerve transfer surgery is the matching of the original dominant muscles of the donor's nerve and the recipient nerve. Since cerebral control of the donor nerve differs from that of the recipient nerve, volitional movement of muscles dominated by the injured nerve after nerve transfer could be restricted and prognosis may be limited by cerebral plasticity (17). In our strategy, both PDIC7 and C5 were initially targeted to the deltoid, which avoided the cerebral function transfer phase of voluntary movement.

In addition, there are other factors that contribute to different outcomes of neurogenic shoulder function improvement, such as the interval between injury and surgery (18), age, and BMI (19), thus, future studies should examine these factors in greater detail. In our study, we applied PDIC7 transfer to the C5 root in patients aged 16–63 years and the interval between injury and surgery was 1–9 months. Most of them achieved satisfied prognosis without related side effects observed during the follow-up. We may, therefore, preliminarily demonstrate that PDIC7 transfer is a safe, one-stage, and effective surgical procedure for patients with neurogenic shoulder abduction limitation. Furthermore, based on our clinical experience, patients with C5 injury who received only PDIC7 transfer can achieve satisfactory improvement in shoulder function without combining other additional surgical strategies.

In our study, the causes behind shoulder abduction limitation varied. Among all, traffic accidents were the most common cause (51.3%) and mechanical injuries accounted for 92.3% of all causes. Also, there were two neuritis cases and one postoperational injury case that underwent our strategy and received a good prognosis. As for patients with neuritis or other neurologic problems, our strategy can also be available for those who did not receive satisfying functional improvement after conservative therapies such as waiting, medicines, or physical therapies. It should be noted that the two neuritis cases in this study had received simple neurolysis surgery but had not obtained satisfying recovery more than 12 months after surgery, thus, we performed a PDIC7 transfer for them and they received benign effects.

Furthermore, the patient whose shoulder abduction only achieved 85° was a 47-year-old male patient. There may be two main reasons for his poor recovery. One is that the injured side was not his dominant side and the other is the long interval between injury and surgery. Before his surgery, there had been apparent muscle atrophy on his injured upper extremity. This question is worth discussing and studying if the long interval time and irreversible muscle atrophy may predict a poor prognosis and the probable degeneration of nerve or muscle tissue during the interval time should be studied more, which is of great clinical value to choosing better treatment strategies for patients.

Although in our study, PDIC7 transfer to C5 has shown a better therapeutic effect than traditional surgical strategies, we still hope to explore more for improvement. For further research, we are curious about if PDIC7 could be divided into more selective branches, which requires not only clinical but also more basic and anatomical studies to get a better understanding of the brachial plexus nerves.

For all patients with neurogenic shoulder abduction limitation, PDIC7 transfer is a generally safe, one-stage, and effective strategy for restoring shoulder function.

## Data availability statement

The original contributions presented in the study are included in the article/supplementary material, further inquiries can be directed to the corresponding author.

## Ethics statement

Ethical review and approval was not required for the study on human participants in accordance with the local legislation and institutional requirements. Written informed consent from the patients/participants or patients/participants' legal guardian/next of kin was not required to participate in this study in accordance with the national legislation and the institutional requirements. Written informed consent was obtained from the individual(s) for the publication of any potentially identifiable images or data included in this article.

## Author contributions

XH and ZY collected the cases and analyzed the data. ZY and JD reviewed the previous literatures. XH and YX wrote the draft. YX and JD revised the manuscript. JJ conceptualized the main idea and supervised the whole study. XH, ZY, YX, JD, and JJ contributed to review the manuscript. All authors contributed to the article and approved the submitted version.

## References

[1] Hsueh YH Tu YK. Surgical reconstructions for adult brachial plexus injuries. Part I: Treatments for combined C5 and C6 injuries, with or without C7 injuries. Injury. (2020) 51:787–803. 10.1016/j.injury.2020.02.076 32156416

[2] Thompson SE Smith ZA Hsu WK Nassr A Mroz TE Fish DE. C5 Palsy after cervical spine surgery: a multicenter retrospective review of 59 cases. Global Spine J. (2017) 7:64s–70s. 10.1177/2192568216688189 28451494 PMC5400195

[3] Suzuki K Doi K Hattori Y Pagsaligan JM. Long-term results of spinal accessory nerve transfer to the suprascapular nerve in upper-type paralysis of brachial plexus injury. J Reconstr Microsurg. (2007) 23:295–9. 10.1055/s-2007-985205 17973214

[4] Leechavengvongs S Witoonchart K Uerpairojkit C Thuvasethakul P Malungpaishrope K. Combined nerve transfers for C5 and C6 brachial plexus avulsion injury. J Hand Surg Am. (2006) 31:183–9. 10.1016/j.jhsa.2005.09.019 16473676

[5] Dong Z Zhang CG Gu YD. Surgical outcome of phrenic nerve transfer to the anterior division of the upper trunk in treating brachial plexus avulsion. J Neurosurg. (2010) 112:383–5. 10.3171/2009.4.JNS081064 19445569

[6] Anastakis DJ Malessy MJ Chen R Davis KD Mikulis D. Cortical plasticity following nerve transfer in the upper extremity. Hand Clin. (2008) 24:425–44, vi–vii. 10.1016/j.hcl.2008.04.005 18928891

[7] Gu YD Cai PQ Xu F Peng F Chen L. Clinical application of ipsilateral C7 nerve root transfer for treatment of C5 and C6 avulsion of brachial plexus. Microsurgery. (2003) 23:105–8. 10.1002/micr.10113 12740881

[8] Xu JG Wang H Hu SN Gu YD. Selective transfer of the C7 nerve root: an experimental study. J Reconstr Microsurg. (2004) 20:463–70. 10.1055/s-2004-833503 15356768

[9] Yin HW Jiang S Xu WD Xu L Xu JG Gu YD. Partial ipsilateral C7 transfer to the upper trunk for C5-C6 avulsion of the brachial plexus. Neurosurgery. (2012) 70:1176–81. 10.1227/NEU.0b013e3182400a91 22072135

[10] Wolfe SW Hotchkiss RN Pederson WC Kozin SH Cohen MS. Green's Operative Hand Surgery. Seventh ed. Philadelphia, PA: Elsevier (2017).

[11] Wei LU Jiang-Guang XU Xiao JD. A study of the motor fiber counts of the human 7th cervical nerve root and its branches. Chin J Clin Anat. (2004) 2004:518–21. 10.13418/j.issn.1001-165x.2004.05.022

[12] Lu W Xiao J Xu J He Q Li J Wang D. [Study on the quantity and distribution of motor fiber of rat's C7 nerve root]. Zhongguo Xiu Fu Chong Jian Wai Ke Za Zhi. (2005) 19:857–9. 16334227

[13] Niu J Li Y Zhang L Ding Q Cui L Liu M. Cross-sectional area reference values for sonography of nerves in the upper extremities. Muscle Nerve. (2020) 61:338–46. 10.1002/mus.26781 31837161

[14] Bedewi MA Kotb MA. Ultrasound reference values of C5, C6, and C7 brachial plexus roots at the interscalene groove. Neurol Sci. (2021) 42:2425–9. 10.1007/s10072-020-04836-1 33074450

[15] Won SJ Kim BJ Park KS Kim SH Yoon JS. Measurement of cross-sectional area of cervical roots and brachial plexus trunks. Muscle Nerve. (2012) 46:711–6. 10.1002/mus.23503 23055312

[16] Hu CH Chang TN Lu JC Laurence VG Chuang DC. Comparison of surgical strategies between proximal nerve graft and/or nerve transfer and distal nerve transfer based on functional restoration of elbow flexion: a retrospective review of 147 patients. Plast Reconstr Surg. (2018) 141:68e–79e. 10.1097/PRS.0000000000003935 29280873

[17] Dahlin LB Andersson G Backman C Svensson H Björkman A. Rehabilitation, using guided cerebral plasticity, of a brachial plexus injury treated with intercostal and phrenic nerve transfers. Front Neurol. (2017) 8:72. 10.3389/fneur.2017.00072 28316590 PMC5334286

[18] Solla DJF de Oliveira AJM Riechelmann RS Martins RS Siqueira MG. Functional outcome predictors after spinal accessory nerve to suprascapular nerve transfer for restoration of shoulder abduction in traumatic brachial plexus injuries in adults: the effect of time from injury to surgery. Eur J Trauma Emerg Surg. (2020). 10.1007/s00068-020-01501-2 32980882

[19] Xiao C Lao J Wang T Zhao X Liu J Gu Y. Intercostal nerve transfer to neurotize the musculocutaneous nerve after traumatic brachial plexus avulsion: a comparison of two, three, and four nerve transfers. J Reconstr Microsurg. (2014) 30:297–304. 10.1055/s-0033-1361840 24683138

